# Incisional hernia repair in rats: description of the sublay technique under videomagnification system

**DOI:** 10.1590/acb370802

**Published:** 2022-10-28

**Authors:** Daniela Ferreira Tramontin, Luís Vinícius Pires da Costa, Nayara Pontes de Araújo, Deivid Ramos dos Santos, Rafael Silva Lemos, Renan Kleber Costa Teixeira, Edson Yuzur Yasojima

**Affiliations:** 1 Graduate student. Universidade do Estado do Pará – School of Medicine – Belém (PA), Brazil.; 2 MD, MS. Universidade do Estado do Pará – School of Medicine – Department of Experimental Surgery – Belém (PA), Brazil.; 3 PhD, Associate Professor. Universidade do Estado do Pará – School of Medicine – Department of Experimental Surgery – Belém (PA), Brazil.

**Keywords:** Abdominal Hernia, Incisional Hernia, Peritoneum, Animal Models, Wistar Rats

## Abstract

**Purpose:**

To describe the technique of sublay correction of incisional hernia in Wistar rats under videomagnification system.

**Methods:**

Five male rats of the species Rattus norvegicus, of the Wistar lineage, with body weight between 250–350 g and 60 days old were used. Incisional hernia was inducted in all animals. After that, the incisional hernia was immediately corrected by the sublay method.

**Results:**

There were no cases of recurrence of the incisional hernia after placement of the polypropylene mesh using the sublay technique. No postoperative complications were observed.

**Conclusions:**

The technique is suitable for execution in Wistar rats.

## Introduction

Incisional hernia is one of the main morbidity factors after abdominal surgery^1^. The incidence is approximately 20% in patients, reaching 40% in high-risk patients^2^. Thus, surgical approaches used for treatment are advancing, and among them, the use of prophylactic meshes can be highlighted, as they are routinely used and prove to be advantageous in terms of results^3^^–^ 5.

To allow the best results, several techniques have been described in humans for the placement of mesh, such as onlay, sublay, preperitoneal plane and intraperitoneal plane, besides the combination of different sites^6^^,^ 7. In addition, the placement of this prophylactic mesh appears to be effective regardless of location, being a safe technique that prevents an increased risk of seroma and chronic pain^4^.

In laparoscopic surgeries, intraperitoneal onlay mesh repair seems to be the only option^8^, but it also appears to be an alternative to open surgeries, as the placement of the mesh intraperitoneally can avoid extensive tissue dissection and reduce the chance of infection at the surgical site and prevent the occurrence of infection through the mesh^9^^–^ 11. Furthermore, this reduction in infection is important because it leads to significant morbidity and mortality, hernia recurrences, prolonged hospital stay and increased hospital costs^12^.

Concerning open surgeries, the sublay technique is the most used^7^, being safe and efficient in patients with more relevant conditions where the surgery should not be contraindicated, despite the risk of progression of the hernia defect and persistent symptoms^13^. In the long term, its repair is superior to the onlay mesh repair, which is technically easier, nonetheless, provides more postoperative complications, not presenting divergence in terms of recurrence rate^14^.

However, there are still disagreements about the most effective method to approach the ideal positioning of the mesh between the layers of the abdominal wall^7^, since each method has its advantages, disadvantages, and variable results according to the literature. Thus, there is a need for comparative studies between the various techniques.

In addition, bringing this context of incisional hernia to the experimental models, it is believed that rats (*Rattus norvegicus*) constitute a suitable animal model for hernia research^15^ with an appropriate anatomical structure for the elaboration of surgical procedures^16^, in addition to presenting low cost and ease of reproduction, it also has great genetic similarity to humans^17^. However, although already described in humans, no articles were found about the use of the sublay technique in Wistar rats, possibly due to the small size of these animals and the difficulty of performing the technique.

Therefore, the objective of this work is to describe the technique of sublay correction of incisional hernia in Wistar rats under videomagnification system, since there is no standardization of this technique in an experimental model.

## Methods

This study followed the principles established by the Brazilian laws of use and creation of animals (No. 11,794/2008), which regulates research with animals in Brazil, being approved by the Ethics Committee in the Use of Animals of the UEPA under the opinion No. 06/2021.

This is an experimental, prospective and descriptive study developed by the Experimental Surgery Laboratory, UEPA. Five male rats of the *R. norvegicus* species, of the Wistar strain, with body weight between 250–350 g and age of 60 days were used. The animals were placed in a vivarium with adequate conditions of temperature (21–24 °C), luminosity (interleaving between light and dark every 12 h), humidity (70–80%) and noise. Furthermore, they were kept in individual cages with sterile wood shavings and received water and food *ad libitum*.

Incisional hernia was induced in all animals, based on the model described by Paulo *et al*.^18^. After that, the incisional hernia was immediately corrected by the sublay method, adapted from the surgical procedure performed in humans described by Miranda *et al*.^19^.

### Videomagnification system

The magnification system^20^^,^ 21 used in this study consisted of a Sony Handycam HDR-XR160 camera connected to a 55’ Full HD Curve TV through an HDMI cable, allowing a magnification of 50× the original size. Two fluorescent light sources were used next to the board to provide adequate illumination of the operative field.

### Anesthetic procedures

The surgical team consisted of a senior surgeon and an assistant surgeon.

The animals were submitted to general anesthesia, by association of 75–100 mg/kg of ketamine hydrochloride and 5–10 mg/kg of xylazine hydrochloride, intraperitoneally. The anesthetic level was confirmed by testing the caudal reflex, foot reflex and vibrissa movement.

Infraumbilical and supraumbilical manual epilation of the anterior abdominal wall was performed in the form of a 6 × 4 cm rectangle. Antisepsis was performed with 0.5% alcoholic chlorhexidine. The animal was fixed on a surgical board in the supine position.

### Operating procedure

#### Induction of incisional hernia

**Figure 1:**
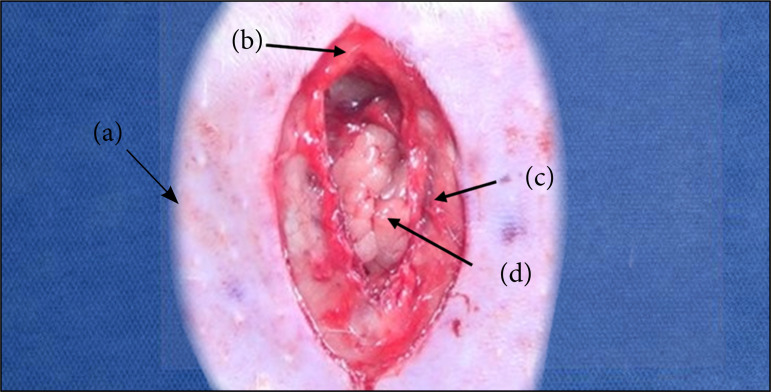
Median incision of 4 cm in the skin and longitudinalincision of the linea alba and peritoneum for an extensionof 3 cm. The structures observed from the most superficialto the most profound are: **(a)** Skin; **(b)** Subcutaneouscell tissue; **(c)** Aponeurosis; **(d)** Abdominal cavity.

The first step of the process consisted of making a median incision of 4 cm in the abdomen below the xiphoid process, using a #15 scalpel blade. Divulsion of the subcutaneous cell plane were performed for approximately 1.5 cm on each side of the linea alba, with the aid of the noncutting face of Metzenbaum scissors. To protect the intraperitoneal viscera, the peritoneum was clamped by two hemostatic forceps, which were pulled, generating a fold, on which a small incision was made, facilitating the removal of the viscera in relation to the serosa. A closed instrument was introduced and it was rotated 360°, undoing possible adhesions of intestinal loops. Then, a longitudinal incision of the linea alba and the peritoneum was made for an extension of 3 cm (Fig. 1).

#### Correction with the sublay method

**Figure 2:**
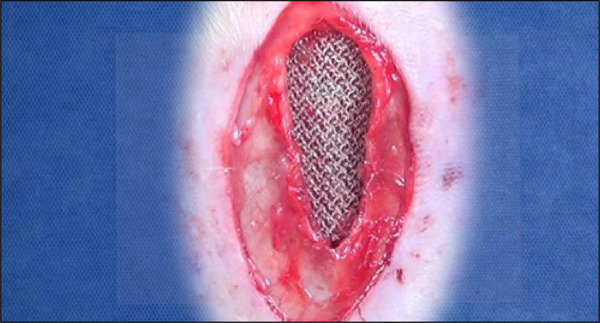
Manual placement of the 3.5 × 2.5 cm polypropylenemesh directly under the rectus abdominis muscle.

**Figure 3:**
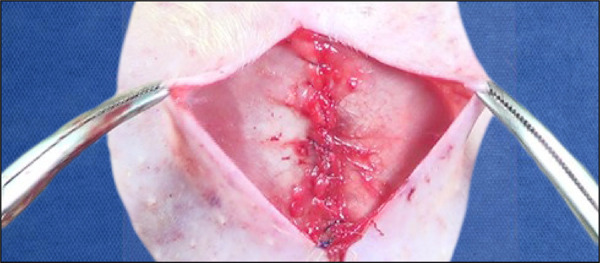
Approximation of the rectus abdominismuscle sheaths by means of continuoussuture with Prolene 5-0 thread.

**Figure 4:**
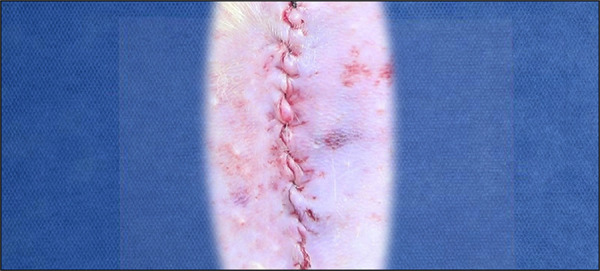
Synthesis of the skin with simpleovercast using the Monocryl thread.

The peritoneal incision was followed by the opening of the medial posterior lamina of the rectus abdominis muscle sheath bilaterally, and a wide dissection was performed between it and the muscle with the aid of the videomagnification system and microsurgical forceps and scissors. This was followed by manual placement of the 3.5 × 2.5 cm polypropylene mesh directly under the rectus abdominis muscle (Fig. 2). The sheaths were brought together through continuous suture with Prolene 5–0 thread (Fig. 3). Skin synthesis was performed with simple stitches at a distance of 1 cm using Monocryl 4-0 thread (Fig. 4).

### Postoperative procedures

After the surgery, the vital signs of the animals were monitored. In addition, sodium dipyrone for veterinary use was administered subcutaneously in doses of 160 mg/kg if the animal showed signs of pain, such as piloerection, hunched posture, aggressive behavior and changes in food and water consumption. In the postoperative period, the animals were housed in individual cages.

### Assessment and euthanasia

The presence of postoperative complications and the occurrence of herniation after 28 days were evaluated, verified by inspection and local palpation.

After 28 days of surgery, the animals were euthanized by intraperitoneal anesthetic overdose, in which triple the dose of the anesthetic protocol was used.

## Results

During the procedure, none of the animals died or required anesthetic boost. The sublay technique proved to be viable for performance in Wistar rats. The mean operative time was 53.4 ± 3.68 min.

There were no cases of recurrence of incisional hernia after placement of the polypropylene mesh by the sublay technique. No postoperative complications were observed, such as hematomas, tissue necrosis, stitch dehiscence or systemic signs and/or sites of inflammation of the surgical wound.

## Discussion

The use of meshes for correction of incisional hernias has provided the use of several surgical approaches for their ideal positioning, in order to avoid increased intraperitoneal pressure and maintain respiratory mechanics at adequate levels. However, there are scientific barriers regarding the effectiveness of each one of them^6^. In this sense, experimental studies become important tools to aid in the development of more effective techniques^23^. However, despite using rats in these studies, there is still no standardization regarding the surgical technique to be performed in these animals to correct this pathology.

Thus, the present study aimed to develop a new technique of retromuscular sublay in rats for the correction of incisional hernia. By the sublay technique, the mesh can be preperitoneal, intraperitoneal or retromuscular^24^^,^ 25. Despite being preferred among surgeons for hernia repair because it provides a low recurrence rate, this technique remains controversial, due to the fragility of the abdominal wall to sustain internal pressure^14^^,^ 22. Thus, the technique developed allows the restoration of the posterior sheath unit of the rat’s rectus abdominis muscle with suture and, after tissue divulsion, deposits the mesh between the fibers of the same and the sheath itself, resulting in greater wall resistance.

According to the data collected, there were no complications such as tissue necrosis, stitch dehiscence and recurrence during the analyzed period of the study. Such complications are frequent after correction of the pathology with the use of meshes, given that it is a foreign body and tissue rejection may occur^9^^,^ 10 ^,^ 26. Even so, the use of this technique in experimental models allows a more in-depth study of the mechanisms that involve failures in the surgical treatment of incisional hernias in humans, due to their genetic and tissue biocompatibility with *R. norvegicus*, a Wistar strain used in most simulators, being able to improve it, minimizing the risks^15^^,^ 17.

However, despite having shown promising results, the technique described has a high degree of difficulty to be performed, since the animal has dimensions much smaller than the human^15^^,^ 17 ^,^ 23 ^,^ 26 ^,^ 27. This fact imposes the need for detailed skills on the part of the surgeon to dissect the fibers without injuring the posterior sheath, otherwise the error in the applied method may result in interpretations that are not consistent with reality. In order to reduce the dimension barrier, the videomagnification system described by Barros *et al.* 21 was used in the study, which has the main advantages of low cost compared to conventional laboratory microscopes and the possibility of magnification of 50× the original size.

In addition, it is worth noting that the study had some limitations. Incisional hernias may recur even after a certain postoperative period^14^. Although the results presented here demonstrate that there was no recurrence by the technique used, this statement is valid only for the analyzed period, and it is not possible to extrapolate this information to longer periods. Added to this, it was not possible to compare the new technique with meshes made of other types of materials, requiring further studies to better evaluate its effectiveness. Even so, the technique described proved to be viable for use in experimental studies.

## Conclusion

The technique is suitable for performance in Wistar rats, proving to be safe, since there were no cases of recurrence of incisional hernia after the placement of the polypropylene mesh by the sublay technique. No postoperative complications were observed. However, it is difficult to perform due to the reduced dimensions of the animals.
